# A cross-sectional investigation of regional patterns of diet and cardio-metabolic risk in India

**DOI:** 10.1186/1475-2891-10-12

**Published:** 2011-01-28

**Authors:** Carrie R Daniel, Dorairaj Prabhakaran, Kavita Kapur, Barry I Graubard, Niveditha Devasenapathy, Lakshmy Ramakrishnan, Preethi S George, Hemali Shetty, Leah M Ferrucci, Susan Yurgalevitch, Nilanjan Chatterjee, KS Reddy, Tanuja Rastogi, Prakash C Gupta, Aleyamma Mathew, Rashmi Sinha

**Affiliations:** 1 Division of Cancer Epidemiology and Genetics, National Cancer Institute, (6120 Executive Blvd), Rockville, MD, (20852), USA; 2 Centre for Chronic Disease Control, (C1/52, Safdarjung Development Area), New Delhi, (110 016), India; 3 Steno Diabetes Center, (Niels Steensens vej 8), Gentofte, (DK 2820), Denmark; 4 Department of Cardiac Biochemistry, All India Institute of Medical Sciences, (Ansari Nagar), New Delhi, (110029), India; 5 Regional Cancer Center, (Medical College Campus), Trivandrum, Kerala (695011), India; 6 Healis Sekhsaria Institute for Public Health, (Thane, Sector 11, CBD Belapur), Navi Mumbai, (400 614), India; 7 Westat, (1600 Research Blvd), Rockville, MD, (20850), USA; 8 UN World Food Programme, (Via Cesare Giulio Viola, 68), Rome, (00148), Italy

## Abstract

**Background:**

The role of diet in India's rapidly progressing chronic disease epidemic is unclear; moreover, diet may vary considerably across North-South regions.

**Methods:**

The India Health Study was a multicenter study of men and women aged 35-69, who provided diet, lifestyle, and medical histories, as well as blood pressure, fasting blood, urine, and anthropometric measurements. In each region (Delhi, n = 824; Mumbai, n = 743; Trivandrum, n = 2,247), we identified two dietary patterns with factor analysis. In multiple logistic regression models adjusted for age, gender, education, income, marital status, religion, physical activity, tobacco, alcohol, and total energy intake, we investigated associations between regional dietary patterns and abdominal adiposity, hypertension, diabetes, and dyslipidemia.

**Results:**

Across the regions, more than 80% of the participants met the criteria for abdominal adiposity and 10 to 28% of participants were considered diabetic. In Delhi, the "fruit and dairy" dietary pattern was positively associated with abdominal adiposity [highest versus lowest tertile, multivariate-adjusted OR and 95% CI: 2.32 (1.03-5.23); P_trend _= 0.008] and hypertension [2.20 (1.47-3.31); P_trend _< 0.0001]. In Trivandrum, the "pulses and rice" pattern was inversely related to diabetes [0.70 (0.51-0.95); P_trend _= 0.03] and the "snacks and sweets" pattern was positively associated with abdominal adiposity [2.05 (1.34-3.14); P_trend _= 0.03]. In Mumbai, the "fruit and vegetable" pattern was inversely associated with hypertension [0.63 (0.40-0.99); P_trend _= 0.05] and the "snack and meat" pattern appeared to be positively associated with abdominal adiposity.

**Conclusions:**

Cardio-metabolic risk factors were highly prevalent in this population. Across all regions, we found little evidence of a Westernized diet; however, dietary patterns characterized by animal products, fried snacks, or sweets appeared to be positively associated with abdominal adiposity. Conversely, more traditional diets in the Southern regions were inversely related to diabetes and hypertension. Continued investigation of diet, as well as other environmental and biological factors, will be needed to better understand the risk profile in this population and potential means of prevention.

## Background

The Indian population has the highest prevalence of diabetes worldwide [1] and exhibits high-risk metabolic profiles at younger ages and lower body mass index (BMI) than their Western counterparts [2,3]. Although genetic susceptibility is likely to play a role in chronic disease etiology, the strong evidence for diet and other environmental factors [4-7] suggest that such an epidemic may be preventable.

Diets across India have not been widely investigated, yet many believe that India may be in the midst of a "nutrition transition," [8-10] where changes in diet parallel an expanding industrial economy and a rapidly progressing epidemic of obesity and chronic, non-communicable disease. In this emerging at-risk population, the suspected access to and adoption of a less healthy diet and lifestyle, and/or deviance from traditional and potentially protective behaviors [9,11,12], may be linked to anthropometric factors and biological markers of chronic disease risk [3,13]. However, few have closely examined the role of diet in these patterns and relationships across a large, diverse, Indian population.

Large cohort studies in the U.S. and Europe have used dietary patterns analyses to bring to light common eating behaviors and their relationships with risk of cardiovascular disease and cancer [14,15]; however, no such studies exist in India. Furthermore, diet is expected to vary considerably within India across North-South regions [10,13,16] and with the exception of national food surveys [9,17], few comprehensive and up-to-date assessments of regional Indian diets are currently available [11,12,18].

Utilizing detailed diet histories collected in a cohort feasibility study conducted across three diverse regions of India, our objective was to aggregate foods in to regional dietary patterns and to investigate associations with cardio-metabolic risk factors, such as abdominal adiposity, hypertension, diabetes, and dyslipidemia. As little is known regarding regional differences, we used an exploratory approach, factor analysis, to empirically identify dietary patterns reflecting actual eating behaviors within each of the study regions [19,20].

## Methods

### Study Participants

**Figure 1:**
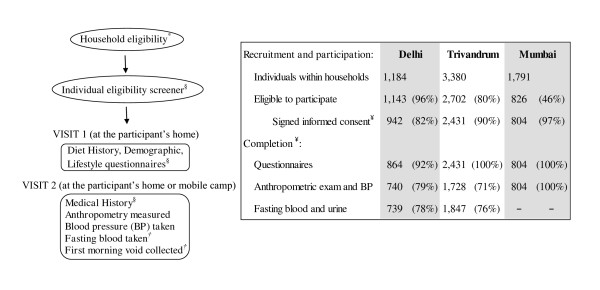
**India Health Study (IHS): Design, visit flow, and participation rates**. *Participant's household selection was stratified by religion, as well as urban-rural status in Trivandrum only. Both Delhi and Mumbai centers are considered to be entirely urban. Religious groups are not evenly distributed throughout the country; therefore, higher concentrations of Muslim and Christian households were recruited from Trivandrum. ^§^All questionnaires were administered by trained field staff via interview. ^†^In Mumbai, no biological samples were collected and all information was collected in the first visit. ^¥^Denominator for completion rates

The India Health Study (IHS; Figure 1) was a multicenter pilot study designed to investigate the feasibility of establishing a diet and cancer cohort in India. The study was conducted between December 2006 and July 2008 in participating centers distributed across three regions of India: Delhi in the north (All India Institute of Medical Sciences and Centre for Chronic Disease Control), Mumbai in the west (Healis-Sekhsaria Institute for Public Health), and Trivandrum in the south (Regional Cancer Center). Centers were selected in areas with established cancer registries [21] and to capture a range of different economic, ethnic, and urbanization patterns [11]. Human ethics committees from each study center, the Special Studies Institutional Review Board of the U.S. National Cancer Institute, as well as the Indian Health Ministry Screening Committee (part of the Indian Council of Medical Research reporting to the Government of India) reviewed and approved the study.

Households within the centers' coverage areas were identified using census data in Delhi, the voter's registration list in Trivandrum, and an existing cohort [22] database of participants and their neighbours in Mumbai. The IHS households were selected at random from census enumeration blocks in 2 wards in the Hauz Khas subdivision of the South District in Delhi; polling stations in 6 urban and 49 rural wards from the Ned umangadu and Thiruvanthapuram taluks (subdivisions) in Trivandrum; and from 3 representative areas (Parel, Naigaum, Sewri) of Ward F-South in Mumbai [22]. In the largest study region, Trivandrum, households were sampled by religion (Hindu/Muslim/Christian) and urban-rural residence strata to maximize the variability in diet and lifestyle across the IHS. Household eligibility was verified by field interviewers during the first in-home recruitment visit.

Individuals within households were eligible to participate if they met the following criteria: 35 to 69 years of age; resided in the study area for a minimum period of 1 year; no prior history of cancer, recent cardiac event, or blood disorders; proficient in English or regional language; capable of informed consent; willing to provide biological samples (feasibility component of pilot cohort); and no physical ailments or limitations preventing them from participating in the study. Eligible female participants could not be pregnant. To achieve a diverse population with variation in diet and other risk factors, one male and one female adult per household were recruited to obtain an approximately equal number of subjects by gender and five-year age category.

From 7,064 households initially identified (Delhi 1,298; Trivandrum 4,915; Mumbai 851), 3,033 (43%) were successfully contacted. Region-specific rates varied due to different methods of identification and recruitment: Delhi 626 (48%); Trivandrum 1,720 (34%); and Mumbai 687 (81%). From these households, 6,355 individuals aged 35-69 were identified and 4,177 were found to be eligible and willing to participate (Figure 1). In the present analyses, participants (Figure 1) were excluded from entering the factor analysis if they reported less than 15 total food items in the Diet History (n = 27, 142, 37 in Delhi, Trivandrum, and Mumbai, respectively) or had missing or invalid responses to the medical history questionnaire (n = 13, 42, 24). Thus dietary pattern values were available for 824, 2,247, and 743 participants. For the purposes of the abdominal adiposity analysis (described in detail below), an additional 121, 458, and 20, were excluded due to missing or implausible height, weight, waist or hip circumference measurements. Across the regions, blood pressure measurements were available for 705 (Delhi), 1,843 (Trivandrum), and 737 (Mumbai) participants to validate medical history data for hypertension (described in detail below). In Delhi and Trivandrum, respectively, 701 and 1,779 participants had valid fasting plasma glucose levels, while an additional 6 and 115 participants had urine sugar values only. Thus, for 707 and 1,894 participants in these two regions we used biological markers to validate diabetes status (described in detail below). In the Mumbai study center, the infrastructure necessary for immediate storage and processing of biospecimens was inadequate and prohibitive; thus, no blood or urine was collected from Mumbai participants.

### Data Collection

Questionnaires and diet assessments were administered via interview by trained field staff in the participants' homes. Socio-demographic, household, diet, physical activity and other lifestyle information, including tobacco and alcohol use, were collected upon enrollment. Anthropometric measures, medical histories, current medications, and biological samples were collected at subsequent visits by medical staff in the participant's home or mobile clinics set up within neighborhoods (Figure 1). In Delhi and Trivandrum, fasting blood, toenail clippings, and first morning void urine specimen were collected at Visit 2. No biological samples were collected in Mumbai due to the lack of proper storage and laboratory resources in the study region.

#### Assessment of Diet

Diet was assessed using a computer-based, interviewer-administered, meal-based, comprehensive diet assessment known as the New Interactive Nutrition Assistant- Diet in India Study of Health (NINA-DISH). This software was developed specifically for the IHS by adapting and modifying software originally developed by Novo Nordisk Pharma India (Bangalore, India). The Diet History (DH) component included three sections: a set of defined questions similar to a food-frequency questionnaire (FFQ); an open-ended section for each mealtime to collect additional and unique regional foods; and a food-preparer's questionnaire. To help participants estimate portion sizes, interviewers provided standard food models along with typical serving plates, bowls, and utensils. The NINA-DISH database included a selection of 910 food items over eight meal times (bed tea, breakfast, mid-morning snack, lunch, afternoon tea, evening snack, dinner and bed-time snack). Participants completed the DH within 20 minutes to 1 hour; and across the three regions reported a total of 576 unique food items (212, 316, and 339 foods in Delhi, Trivandrum, and Mumbai, respectively).

There was no single up-to-date and comprehensive Indian food and nutrient database currently available for estimating nutrient values from regional Indian foods [11]; thus, we pooled the relevant data from existing international databases containing Indian foods and recipes. Nutrient values and recipe codes were drawn from the U.K.'s Nutrient Databank/McCance and Widdowson's The Composition of Foods integrated dataset (CoF IDS), the U.S. Department of Agriculture (USDA)'s Food and Nutrient Database for Dietary Studies (FNDDS), the Singapore Health Promotion Board (HPB)'s Food Info, the Malaysian Foods Composition Database (NutriWeb), the Australian Food Composition Tables (NUTTAB), and the Food and Agriculture Organization of the United Nations (FAO)'s World Food Dietary Assessment System (WorldFood). For items that could not be matched, recipes were developed using a combination of database-matched cooked foods. For each nutrient, we used a standardized unit of measurement and reported values per 100 grams of edible portion of food product [23,24].

#### Blood Pressure, Anthropometry, and Physical Activity

At the time of the anthropometry exam, systolic and diastolic blood pressure levels were taken by a study nurse using a portable calibrated digital monitor for the upper arm. Using a portable calibrated digital scale (Breuer), stadiometer (Seca 214), and no-stretch measuring tape (Gulick), weight, height, waist circumference, hip circumference, and thigh circumference were taken in duplicate (or triplicate if the first two were not within agreement) and then averaged. Body mass index (BMI) was calculated as weight (kg)/height (m)^2 ^and categories were based on World Health Organization cut-points with special consideration of Asian populations [25]. Abdominal adiposity was estimated with the waist circumference to hip circumference ratio (WHR) [26]..

All study participants completed the validated short-form of the International Physical Activity Questionnaire [IPAQ; [27,28]]. We estimated total physical activity as the weighted sum of walking, moderate, and vigorous activity in metabolic equivalents of task (MET)-hours per week from recreation, occupation, household work, and transportation-related activity [29].

#### Biospecimen collection and processing

In Delhi and Trivandrum, a first morning urine sample (100 mL) and fasting blood sample (15 mL) were collected and immediately transported (within three hours) to the respective center's clinical laboratories in coolers for routine processing and analysis using a Beckman Autoanalyzer (Beckman, Fullerton, CA). Fasting glucose levels were determined with the glucose oxidase/peroxidase method [30] (Delhi: GOD-PAP; Randox Laboratories Ltd., Antrim, UK; Trivandrum: GOD-POD; Autospan; Span Diagnostics Ltd., Surat, India). In Delhi, lipid profiles were also analyzed using the following methods: (TC) by cholesterol oxidase/p-aminophenazone (CHOD-PAP; Randox Laboratories Ltd., Antrim, UK) method, triglyceride by glycerolphosphatase oxidase-peroxidase aminophenazone (GPO-PAP; Randox Laboratories Ltd., Antrim, UK) method and HDL cholesterol by precipitation method using phosphotungstate/magnesium-precipitation of apolipoprotein B containing lipoproteins followed by estimation of cholesterol in supernatant by enzymatic method. LDL cholesterol was estimated using the Friedwald formula [31].

#### Prevalent abdominal adiposity, diabetes, hypertension, and dyslipidemia

For the purposes of this analysis, sex-specific cut-points for South Asian [25,26] populations were used to define abdominal adiposity by a WHR greater than or equal to 0.90 in males or 0.80 in females.

Diabetes status was defined by a combination of self-reported medical history, medication use, and fasting plasma glucose levels. Any participants who reported a positive medical history with valid medication use/treatment for high blood sugar were considered diabetes cases; and thus, represented 47% of the total diabetes cases in Delhi and Trivandrum, and 89% of the total cases in Mumbai. Note that in Mumbai, no biospecimens were collected and only self-reported medical history was available to define diabetes status. Of the Delhi and Trivandrum participants who did not report a treatment and/or positive medical history for diabetes, case-status was defined by a fasting plasma glucose level greater than or equal to 126 mg/dl [32]. Fifty-two percent of the total diabetes cases in Delhi and 48% of the cases in Trivandrum were captured by high fasting blood levels; and thus, represented participants who were likely unaware of their diabetic status. Above-normal urine sugar (>15 mg/dl or >0%) was also used to define diabetes status in 1% of the total cases in Delhi and 4% of the total cases in Trivandrum when fasting blood was unavailable. Among non-cases in Delhi and Trivandrum, 91% and 94% were confirmed "normal" by blood levels (<126 mg/dl) or urine, while the remaining 9% and 6% were included as a non-case based on a self-reported negative medical history only. In Delhi and Trivandrum, a variable was similarly created to compare participants with and without pre-diabetes or impaired fasting glucose (≥100 mg/dl versus <100 mg/dl [32,33]).

Hypertensive status was similarly defined in all regions by medical history, medication use, and blood pressure levels. A participant was considered hypertensive if they indicated a positive medical history with valid treatment for high blood pressure; otherwise, hypertension was classified based on blood pressure levels measured in the study (SBP ≥ 140 mmHg or DBP ≥ 90 mmHg). Less than 2% of hypertension cases were based on a positive self-report only. In Delhi, Trivandrum, and Mumbai, respectively, 13%, 8%, and 2% of non-cases were based only on a negative self-reported history.

Blood cholesterol levels were analyzed for Delhi participants only and dyslipidemia was defined by one or more of the following: HDL cholesterol levels less than 40 mg/dl in males or 50 mg/dl in females, TG levels above 200 mg/dl, total cholesterol to HDL ratio greater than or equal to 5 [34].

### Statistical Analysis

We derived dietary patterns for each region separately using factor or principal component analysis (PCA) [20]. Prior to analysis, individual food items collected in the structured DH and in the open-ended section were aggregated in to 130 common "reference groups" by a trained nutritionist familiar with regional food items, recipes, and nutrient content (Table 1). These groups were similar to the level of detail found in a comprehensive FFQ. For example, vegetable items were classified according to their color or botanical group (e.g., red, orange, cruciferous, allium). Next, the reference group data was prepared to enter the factor analysis in a standard manner described elsewhere [19,35]. Very low consumption food groups were either dropped or combined (e.g., Western fast foods) for a total of 104 unique items across all regions. Additional items were dropped in region-specific analyses, as necessary; thus 71, 85, and 81 food items entered the analysis in Delhi, Trivandrum, and Mumbai, respectively. Each of the food items were adjusted for total energy intake using a caloric density approach (each individual's food item frequency was divided by their total energy intake value). Then, by region and gender each energy-adjusted value was standardized to a mean of zero and a standard deviation of one (Z-score). In the initial analysis, each of the energy-adjusted and standardized frequency variables entered the principal components or factor analysis (PROC FACTOR; SAS version 9.2; SAS Institute Inc., Cary, NC) allowing up to six factors per region. Following evaluation of scree plots, eigenvalues, the proportion of explained factor variance, and overall interpretability, we retained the first two components from the initial analysis for each region. In the final analyses limited to two components per region, we rotated each factor using the VARIMAX option in PROC FACTOR to obtain an orthogonal solution. For each subject within a region, we calculated factor scores for each of the rotated factors by summing the frequency of consumption multiplied by the factor loadings across all food items in that region. Mean scores for each of the region-specific primary and secondary factors were categorized into sex-specific tertiles. Thus, for each regional factor or dietary pattern, the highest tertile represents persons whose diets conformed most closely to that particular pattern (highest concordance) and the referent or lowest category represents the lowest concordance. Factor names were defined by carefully examining factor loadings for food items, as well as Spearman correlations for dietary patterns with broader food groups (e.g., total rice, total wheat, total fruit, etc.; Table 1) and macronutrients. We also examined factor analyses in men and women separately, but the dietary patterns were found to be nearly identical to each other and to the combined results presented.

**Table 1:** Food groups and reference groups created from individual food items collected in the comprehensive Diet History, India Health Study

**Food groups**	**Reference groups**	**Food items**
		
Example:	Example of reference groups for rice:	Examples of food items for the reference group "plain rice":
*Rice*	*Plain rice*	*Basmati rice*
	*Fermented rice*	*White-milled rice*
	*Fermented rice dish with*	*Brown rice*
	*Pulses*	*Rice Bhakri*
	*Plain rice dish with pulses*	*Oratti*
	*Mixed rice dish*	*Kanji Rice*
		*Puttu*
		*Idiappam*

Using multiple logistic regression, we investigated cross-sectional associations between dietary patterns and cardio-metabolic markers of risk, including abdominal adiposity, impaired fasting blood glucose (pre-diabetes), dyslipidemia, diabetes, and hypertension. Multivariate models were adjusted for the following covariates (categories defined in Table 2): age (continuous), gender, education, income, religion, marital status, history of tobacco use, history of alcohol use, and physical activity (modeled in region and gender-specific tertiles). We evaluated multivariate models with and without a continuous covariate for total energy intake [36,37]. Inclusion of total energy intake tended to attenuate the effect estimates; thus, the more conservative, adjusted results are presented. If necessary to prevent issues with model fit, variable categories with small cells in some of the regions were collapsed [e.g., religion (Hindu/other), income (high/low), education (high/low), tobacco use (any/none)]. Adjustment for other prevalent chronic conditions (e.g., diabetes models adjusted for hypertension and/or dislipidemia, abdominal adiposity models adjusted for diabetes and/or hypertension, etc.) did not appreciably change the estimates. *P *values for linear trend were estimated by creating a continuous variable using the sex-specific median value within tertiles. We did not find any evidence of effect modification by gender. All statistical tests were considered statistically significant when two-sided *P *< 0.05. All analyses were conducted in SAS version 9.2 (SAS Institute Inc., Cary, NC).

**Table 2:** Characteristics of India Health Study participants who completed Diet and Medical Histories

	Delhi	Trivandrum	Mumbai
Characteristic	*n = 824*	*n = 2,247*	*n = 743*
Age distribution, yrs			
35 to 39	32	21	16
40 to 49	31	33	31
50 to 59	23	29	31
60 to 69	14	17	22
Female,%	53	50	52
Highest education attained,%			
Middle school	34	44	38
Secondary school	27	46	56
University	39	10	5
Married,%	94	93	81
Household monthly income, INR,%			
<5,000	6	69	36
5,000-10,000	18	27	45
>10,000	76	4	19
Primary religion,%			
Hindu	77	34	57
Muslim	3	34	<1
Christian	<1	32	5
Other (Sikh, Buddhist, Jain)	20	<1	38
History of tobacco use			
None	81	73	50
Smokeless (chewing, snuff, betel)	3	5	44
Smoke (cigarettes, bidi, hukka)	16	22	6
History of alcohol use			
No	79	85	79
Yes	21	16	21
Physical activity, MET-hr/wk*	42.8 (45.6)	153.2 (107.9)	179.1 (141.5)
Obesity^†^,%	56	49	46
Abdominal adiposity^§^,%	92	90	86
Dyslipidemia^¥^,%	56	-	-
Diabetes^∫^,%	18	28	10
Medical history	10	17	10
Fasting plasma glucose test ≥ 126 mg/dl	8	11	-
Hypertension^≠^,%	47	51	46
Medical history	18	16	20
SBP ≥ 140 mmHg or DBP ≥ 90 mmHg	29	35	26
Dietary intake,% of total energy*			
Fat	41.1 (4.9)	23.3 (5.8)	39.7 (5.3)
Carbohydrate	48.2 (5.4)	60.5 (6.2)	47.3 (5.4)
Protein	12.1 (1.6)	15.0 (2.2)	13.7 (2.1)

Abbreviations: INR, Indian Rupee; MET, metabolic equivalents of task

All estimates exclude missing data.

* Presented as mean and standard deviation (SD)

^† ^Body mass index ≥ 25 kg/m^2^; WHO universal cutpoint for overweight and suggested Asian-specific cut-point for obesity

^§ ^Waist-to-hip ratio ≥ 0.90 cm in males or 0.80 cm in females

^¥ ^HDL < 40 mg/dl in males or 50 mg/dl in females, triglycerides > 200 mg/dl, total cholesterol to HDL ratio ≥ 5

∫ Positive medical history (self-report) with current medication use or fasting plasma glucose ≥ 126 mg/dl

≠ Positive medical history (self-report) with current medication use or SBP ≥ 140 mmHg or DBP ≥ 90 mmHg

## Results

Table 2 presents the characteristics of the India Health Study participants by region. Delhi had the greatest proportion of participants with higher education and income. Based on BMI alone, approximately half of the participants across the regions were overweight or obese, while 80-90% met the criteria for abdominal adiposity. By gender (data not shown), 4% of women and 6% of men had a BMI < 18.5, while 20% of women and 6% of men had a BMI ≥ 30 [25]. Across the regions, the prevalence of hypertension ranged from 46 to 51% and the prevalence of diabetes ranged from 10 to 28%. In Delhi, participants had the lowest physical activity levels and more than half were dyslipidemic. In Trivandrum, participants appeared to consume a lower proportion of fat and higher proportion of carbohydrate than participants in Delhi or Mumbai. However, the distribution of macronutrients within each region did not vary substantially across tertiles of dietary patterns (Additional file 1: Appendix). Across all regions, concordance with dietary patterns across tertiles of mean factor scores did appear to differ quite markably with levels of physical activity, income, and education (Additional file 1: Appendix).

Table 3 shows the main results of the factor analysis and presents for each region the two retained factors and top loading food items defining each dietary pattern. Table 4 presents correlations between nutrient intakes, broader food groups, and dietary patterns within each region. In Delhi, the primary pattern, which we called "fruit-dairy," included fruit, fruit juice, and mixed dishes likely to contain cheese, yogurt, or other types of dairy. It was most strongly correlated with total fruit (r = 0.55), total dairy (r = 0.51), calcium, and cholesterol intake (r > 0.20). The secondary pattern in Delhi, "vegetables-pulses," was positively correlated with total vegetables (r = 0.46), total pulses (r = 0.32), total fat (r = 0.22), and retinol intake (r = 0.46), but inversely correlated with rice, meat, and protein intake (r < -0.20). In Trivandrum the "pulses-rice" pattern was defined by top loading food items or mixed dishes composed mainly of pulses and fermented rice (Table 3). This pattern was also inversely correlated with intakes of iron, calcium, retinol, and total fish (Table 4). The top loading items for the secondary "sweets-snacks" pattern in Trivandrum were mainly sweet and fried savory snacks (Table 3). This pattern was also positively correlated with fat, cholesterol, and retinol intake (r≥0.37), as shown in Table 4. In Mumbai, the primary "fruit-vegetables" pattern was the most strongly correlated with total vegetables and total fruit (r = 0.72), as well as calcium, iron, and retinol intake. The "snacks-meat" pattern in Mumbai was strongly correlated with intake of cholesterol (r = 0.57), meat (r = 0.46), and sweets (r = 0.32).

**Table 3:** Top 10 loading food items* for primary and secondary factors by region, India Health Study


	**Delhi *(n = 824)***			**Trivandrum *(n = 2,247)***			**Mumbai *(n = 743)***		
	**Food item**	**1°**	**2°**	**Food item**	**1°**	**2°**	**Food item**	**1°**	**2°**

*1*	other white fruit^║^	56	-	pulses with vegetables	64	-	orange vegetables	59	-
*2*	palak paneer/green leafy mixed dish	52	-	tea	61	-	green fruit	57	-
*3*	sandwiches^¶^	52	-	fermented pulse and rice/dosa	52	-	orange fruit	56	-
*4*	pulses with rice/khichri	48	-	other mixed vegetable dishes^‡^	50	-	cauliflower/radish/cruciferous	45	-
*5*	non-citrus fruit juice	46	-	plain rice	45	-50	tea	-44	-
*6*	soups	45	-	chutneys	44	-	curd/yogurt	43	-
*7*	other fruits, local/exotic	43	-	cabbage/green leafy cruciferous	41	-	chutneys	43	32
*8*	chutneys	43	-	other green vegetables^†^	38	-	other white fruit^║^	41	-
*9*	eggplant/purple vegetables	41	-	fermented rice	36	-	gourd vegetables	41	-
*10*	pilaf/mixed rice	40	-	potato	30	-	upma/paratha/wheat with fat	40	-
									
*1*	potato with other green vegetables^†^	-	57	cereal-based sweets^§^	-	46	fried savory snacks, fresh^§^	-	67
*2*	gourd vegetable	-	54	pulse-based sweets^§^	-	43	egg	-	58
*3*	spinach/green leafy vegetables	-	48	fried savory snacks, fresh^§^	-	39	spinach/green leafy vegetables	-	-53
*4*	tea	-	46	red vegetables	-	36	fried savory snacks, dry^§^	-	45
*5*	cauliflower/radish/cruciferous vegetables	-	45	fried savory snacks, dry^§^	-	35	potato with other green vegetables^†^	-	-43
*6*	pulses with skin	-	44	fresh fish	-	-34	eggplant/purple vegetables	-	-40
*7*	fried savory snacks, fresh^§^	-	-44	banana	-	30	potato with cruciferous vegetables	-	-37
*8*	upma/paratha/wheat with fat	-	-42	eggplant/purple vegetables	-	30	pulses with skin	-	-35
*9*	biscuits	-	41				ice cream/kulfi/sorbet	-	32
*10*	pickles	-	-37				nuts	-	31
									

N = 824; 2,247; 743 in Delhi, Trivandrum, and Mumbai, respectively

*Rotated factor pattern; values (correlation coefficients) are multiplied by 100 and rounded to the nearest integer; top factor loadings (≥|30|) for food items are presented; factor loadings <30 are not shown for simplicity

^║^other than banana (e.g., lichi, pear, guava, musk melon, quince)

^¶^not including hamburgers/fast-food, e.g., vegetable, chicken, or cheese sandwiches;

^†^other than leafy or cruciferous (e.g., peas, capsicum, ladies finger)

^‡^vegetable curries and stews (e.g., avial, theeyal)

^§^Indian snacks/sweets: fresh fried savory (e.g., vada, samosa); dry fried savory (e.g., murukku, fruit chips); non-fried savory (e.g., vegetable puff, mince pie, dhokla); cereal-based sweet (e.g., halwa, rice puttu, diwali); milk-based sweet (e.g., custard, khoa); pulse-based sweet (e.g., ladoo, puran poli); fruit/vegetable-based sweet (e.g., carrot/gourd halwa, fried bananas)

**Table 4:** Spearman correlations of the mean factor scores for regional dietary patterns with nutrients and food groups, India Health Study

	Delhi		Trivandrum		Mumbai	
	Factor 1	Factor 2	Factor 1	Factor 2	Factor 1	Factor 2
	*fruit - dairy*	*vegetables - pulses*	*pulses - rice*	*sweets - snacks*	*fruit - vegetables*	*snacks - meat*
Nutrient intake						
protein,% TEI	NS	-0.24	NS	-0.19	-0.11	0.25
carbohydrate,% TEI	0.18	-0.13	-0.25	-0.31	NS	NS
fat,% TEI	-0.18	0.22	0.32	0.48	NS	-0.16
cholesterol, mg	0.24	0.14	-0.19	0.38	0.24	0.57
calcium, mg	0.23	0.09	-0.26	0.34	0.61	0.18
iron, mg	-0.24	0.17	-0.60	0.20	0.55	0.25
retinol equivalents, mcg	-0.08	0.46	-0.28	0.37	0.35	NS
Food group, frequency						
total rice	NS	-0.23	NS	-0.07	0.34	NS
total wheat	-0.08	0.18	-0.12	0.42	0.21	0.20
total vegetables^†^	0.08	0.46	0.08	0.52	0.72	-0.15
total potatoes	0.09	0.08	-0.10	0.10	0.35	NS
total pulses	-0.53	0.32	0.26	0.23	0.35	0.20
total fruit	0.55	0.22	-0.16	0.43	0.72	0.12
total dairy	0.51	-0.09	0.10	0.34	0.51	0.16
total fish	-0.19	-0.21	-0.43	-0.07	0.17	0.32
total meat	0.12	-0.21	-0.20	0.32	0.10	0.46
total sweets	-0.09	-0.10	-0.19	0.50	0.39	0.32

N = 824; 2,247; 743 in Delhi, Trivandrum, and Mumbai, respectively

Abbreviations: TEI, total energy intake; NS, not statistically significant

^†^not including potatoes

All P < 0.01 unless otherwise specified as not significant (NS) for multiple comparisons

Multivariate-adjusted associations for dietary patterns with abdominal adiposity, hypertension, and diabetes within each region are presented in Table 5. In Delhi, the fruit and dairy pattern was positively associated with both abdominal adiposity and hypertension, but not associated with diabetes or dyslipidemia (not shown). No associations were found for the secondary pattern in Delhi. In Trivandrum, the pulses and rice pattern was inversely associated with diabetes (IFG ≥126 mg/dl) and/or pre-diabetes [IFG≥100 mg/dl; OR and 95% CI for highest versus lowest tertile, 0.69 (0.52-0.92); P trend = 0.01; data presented in text only]. The sweets and snacks pattern was positively associated with abdominal adiposity [OR and 95% CI: 2.05 (1.34-3.14); P trend = 0.03] and pre-diabetes [IFG≥100 mg/dl; OR and 95% CI for highest versus lowest tertile, 1.20 (0.94-1.53); P trend = 0.16; data presented in text only], but not associated with diabetes (IFG ≥ 126) or hypertension. In Mumbai, the fruit and vegetables pattern was inversely associated with hypertension, while the snack and meat pattern appeared to be positively associated with abdominal adiposity [OR and 95% CI: 1.61 (0.96-2.71); P trend = 0.09].

**Table 5:** Multivariate-adjusted associations of regional dietary patterns with abdominal adiposity, hypertension, and diabetes status, India Health Study

	Abdominal Adiposity				Hypertension				Diabetes^¥^			
	N_prev_^†^	OR*	95% CI	*P*_*trend*_	N_prev_^†^	OR*	95% CI	*P*_*trend*_	N_prev_^†^	OR*	95% CI	*P*_*trend*_
DELHI	N_tot_^§^=703				N_tot_^§^=763				N_tot_^§^=767			
*Fruit - dairy*												
T1	204	1.00			98	1.00			44	1.00		
T2	218	1.04	0.55-1.97		108	0.93	0.63-1.38		56	1.13	0.71-1.80	
T3	224	2.32	1.03-5.23	*0.008*	153	2.20	1.47-3.31	*<0.0001*	38	0.66	0.39-1.11	*0.07*
*Vegetables - pulses*												
T1	223	1.00			134	1.00			46	1.00		
T2	209	1.14	0.55-2.39		100	0.67	0.46-1.00		47	1.18	0.73-1.89	
T3	214	0.76	0.38-1.53	*0.19*	125	0.90	0.61-1.33	*0.83*	45	0.93	0.57-1.50	*0.65*
												
TRIVANDRUM	N_tot_^§^=1,789				N_tot_^§^=1,932				N_tot_^§^=2,014			
*Pulses - rice*												
T1	558	1.00			325	1.00			193	1.00		
T2	523	0.72	0.45-1.14		311	0.99	0.77-1.27		169	0.74	0.56-0.96	
T3	542	0.88	0.39-1.13	*0.09*	358	1.13	0.84-1.52	*0.41*	205	0.70	0.51-0.95	*0.03*
*Sweets - snacks*												
T1	471	1.00			325	1.00			185	1.00		
T2	559	2.15	1.41-3.29		339	1.12	0.88-1.44		187	1.13	0.87-1.47	
T3	594	2.05	1.34-3.14	*0.03*	330	1.04	0.80-1.34	*0.87*	195	1.19	0.90-1.56	*0.25*
MUMBAI^¥^	N_tot_^§^=723			N_tot_^§^=743								
*Fruit - vegetables*												
T1	198	1.00			129	1.00						
T2	206	1.09	0.67-1.78	111	0.70	0.47-1.05						
T3	220	1.28	0.73-2.26	*0.37*	105	0.63	0.40-0.99	*0.05*				
*Snacks - meat*												
T1	200	1.00			106	1.00						
T2	208	0.97	0.61-1.54		114	1.05	0.71-1.55					
T3	216	1.61	0.96-2.71	*0.09*	125	1.21	0.81-1.82	*0.32*				

Abbreviations: OR, odds ratio; CI, confidence interval; T, tertile

^§^Total number in each region; missing excluded from analysis

^†^Number of participants who have condition within each sex-specific tertile of dietary pattern concordance

*Multivariate adjustment for age, gender, education, income, marital status, tobacco and alcohol use, religion, physical activity, total energy intake

^¥ ^No fasting blood collected in Mumbai

In the largest study region, Trivandrum, we conducted a sensitivity analysis (data presented in text only) comparing dietary patterns associations in participants with normal blood glucose (<100 mg/dl; n = 661; referent group) to each of the following: prediabetics (100-125 mg/dl; n = 407), untreated diabetics (≥126 mg/dl; n = 232), and treated diabetics (valid prescription medication for managing blood sugar; n = 252). The pulses and rice pattern was most strongly inversely associated with treated diabetes [multivariate-adjusted OR and 95% CI, highest to lowest tertile: 0.54 (0.33-0.89); P trend = 0.02]. Conversely, the snack and meat pattern was positively associated with treated diabetes [(1.00 (ref), 1.69 (1.11-2.56), 1.76 (1.12-2.75); P trend = 0.02].

## Discussion

In each of the study regions two dietary patterns emerged and varied associations with cardio-metabolic risk factors were observed. In cross-sectional analyses adjusted for key demographic and lifestyle confounders, diets across all regions characterized by dairy, fried snacks, and sweets appeared to be positively asssociated with abdominal adiposity. Conversly, dietary patterns in Trivandrum and Mumbai, characterized by intake of vegetables and pulses, were inversely related to diabetes and hypertension.

South Asians in India, and throughout the world, are an important population to study due to their greatly elevated risk of diabetes and cardiovascular disease [38,39]. However, compared to the breadth of studies on Western-style dietary patterns and chronic disease etiology [14,40], few studies have examined food patterns in the high-risk Indian population [13,16,18,41-43] or collected biological samples. Compared to Caucasian populations, South Asians typically develop metabolic syndromes at lower BMIs, and are known to have increased visceral fat and insulin resistance [3,44]. Abdominal adiposity, impaired glucose and lipid levels, as well as hypertension in a high-proportion of study participants corresponds with the high-risk "Asian Indian phenotype" that may be a product of genetic adaptations to food insecurity, fetal or early childhood malnutrition, as well as more recent environmental exposures including adult diet [2,18,44-49].

High fat intake (~40% of total energy intake), particularly in Delhi and Mumbai, was one indication that components of the Indian diet may be contributing to a high-risk diet and health profile. However, we found little evidence of Westernization of the diet [50] through food items, such as red meat, sweetened beverages, and processed or fast foods, but did observe other characteristics such as sugary and high-fat foods perhaps reflecting a transitional diet arising from access to cheap oils and sweeteners [8]. Top loading food items for the dietary patterns included fruit, vegetables, chutneys, and tea, as well as traditional Indian fried snacks and desserts. Across all regions, dietary patterns frequently contained traditional mixed dishes composed mainly of vegetables, pulses, cereals and/or potato, but there were regional differences in the types of cereals (e.g., fermented rice, plain rice, wheat products) and potential protein sources such as pulses, dairy, and eggs. A study conducted in the 1970's among Indian physicians [51,52], reported a very low-fat, rice and pulse-based diet in the South. This contrasted with a high-fat, wheat-based diet in the North with frequent consumption of vegetables cooked in ghee, milk, and yogurt. More recently, dietary data collected in India from women in the National Family Health Survey (NFHS-2)[17] also found that intake of animal foods (eggs, dairy, fish, and meat) varied across the different regions [16]. Compared to China and other Asian countries, high amounts of sugar are consumed in India [9]. Our analysis suggests a continued preference for the traditional Indian sweets, as opposed to Western desserts (cake, pies, candy, etc.) which may be more easily recognized as unhealthy [53]. In addition to sugar, many of these Indian sweets are often prepared with a substantial amount of saturated fat from ghee or coconut components.

Previous studies have reported that cardiovascular disease risk in India is likely to be inversely associated with intake of fruits, vegetables, and mustard oil [12,43]; and positively associated with intake of refined carbohydrates and unhealthy fats (reviewed in [18,54]). In Trivandrum we found that the more traditional pulses and rice pattern was inversely associated with diabetes. Similarly, the more prudent-appearing fruit and vegetable pattern in Mumbai was inversely associated with hypertension. Secondary patterns in both of these regions, characterized by intake of fried snacks and sweets, appeared to be positively associated with abdominal adiposity. In Delhi, the northern-most region, where dietary patterns appeared to reflect access to a greater variety of foods, the predominant fruit and dairy pattern was positively associated with both abdominal adiposity and hypertension. Although one may not typically consider fruit part of a high-risk diet, higher concordance with this pattern was more common among participants of higher socio-economic status and lower physical activity and may also reflect dietary choices or substitutions to manage a chronic condition. Unlike Trivandrum and Mumbai, the vegetables and pulses pattern in Delhi, was not associated with any chronic conditions and we found no associations for dietary patterns and dyslipidemia.

The relationship between diet and chronic disease risk is certainly complex and we are likely to encounter unfamiliar challenges in the Indian diet. The type, as well as the cooking, of vegetables (green leafy, starchy, stir-fried, stewed, boiled, etc.) in traditional Indian mixed dishes may alter some of their preventive properties [12,55-57] and may also contribute substantially to added fat [51,58]. The contrasting correlations we observed between intakes of iron, calcium, and retinol and the more traditional patterns in Trivandrum versus Mumbai, suggest that the nutrient quality of these regional diets vary considerably. Other cross-sectional studies in South Asian populations, living in India and abroad, have suggested that characteristically high fruit and vegetable intake may be associated with lower LDL and total cholesterol [43], but that high-carbohydrate diets, overall, may be associated with higher triglycerides and lower HDL cholesterol [59,60], as well as hyperinsulinemia [61] in South Asians. Unraveling the nutrient composition of specific Indian foods [41], particularly the fatty acid, sodium, and glycemic composition [58], as well as the complex relationship between diet, socio-economic status, obesity, and chronic disease risk [62] is likely to be of great relevance for India.

Major strengths of our study were the use of interviewer-administered questionnaires developed specifically for the study, collection of fasting blood, and measurement of anthropometry and blood pressure by trained medical staff. Fasting blood and blood pressure measurements, along with medication use, were invaluable in classifying diabetes and hypertension status, as self-reports were clearly an underestimate of the prevalence in this population. However, the external validity of our findings may be limited in this lower to upper middle class sample with very high rates of abdominal adiposity and obesity. As with many developing countries, a moderate improvement in socio-economic status is likely to increase access to both over-nutrition and a sedentary lifestyle; thus, affluence may serve as a key risk factor for obesity, diabetes, and other related conditions [63]. Other large cross-sectional studies have observed a double burden of under- and over-weight [17,64-66] and throughout India, nutritional status and chronic disease risk profiles vary substantially by intra-and inter-state socio-economic extremes [67-69]. The prevalence of cardio-metabolic risk factors we observed more closely resembled studies of middle-aged adults residing in metropolitan areas of India [70,71] and that of South Asians residing in the U.S. [72]. However, it also plausible that recruitment of IHS participants from households may have resulted in a clustering of risk factors [73].

We recognize the limitations of the cross-sectional study design to make strong conclusions regarding causality and chronic disease etiology. Dietary patterns identified by factor analysis are intended to represent the actual eating patterns that arose from the study sample, but do not necessarily capture optimal diets or unhealthy extremes that one may want to specifically target for intervention or prevention. Factor analysis also involves some level of subjectivity in selecting and grouping food items. However, in our case, a trained nutritionist in India grouped all the food items collected in the DH into meaningful subgroups prior to the patterns analysis. In addition to the number and composition of the food items, various statistical options and methods for factor analysis, as well as the selection of the number of factors to retain in the final analysis, may also affect the overall explained factor variance [74]. Although we used standard methods and *a priori *grouped food items, the detailed DH and large number of food items collected [75] may have limited variation explained by the dietary patterns (8-12% within each region), as well as overall interpretability. Although our variance estimates exceeded those from analyses in similarly underserved populations [76], some larger U.S. cohort studies achieved total explained variance levels as high as 20 to 30% [35,77]. Dietary patterns analysis conducted in some Western cohorts may also have benefitted from a larger sample size, as well as a more cohesive population with regard to ethnicity, education, and economic access to a variety of foods to meet nutritional needs and preferences.

## Conclusions

Participants in all three regions primarily consumed food items that did not appear largely "unhealthy" by Western standards. More traditional dietary patterns in the southern regions, characterized by intake of vegetables and pulses, were inversely associated with the prevalence of diabetes and hypertension. However, overall anthropometric and biological measurements spanning across the majority of the study population characterized high-risk levels not usually seen in studies within the U.S. and Europe. Compelling associations between regional Indian diets and highly prevalent cardio-metabolic risk factors, such as abdominal adiposity and hypertension, suggest that many of the unique regional components of Indian diets, such as high-fat dairy, sweets and fried snacks, may be characterizing a high-risk diet. Continued investigation of diet, evaluating both quality and quantity, as well as other environmental and biological factors, will be needed to better understand the risk profile in this population and potential means of prevention.

## Abbreviations

BMI: body mass index; CI: confidence interval; DH: diet history; FFQ: Food Frequency Questionnaire; IHS: India Health Study; HDL: high density lipoprotein; IFG: impaired fasting glucose; LDL: low density lipoprotein; MET: metabolic equivalents of task; OR: odds ratio; TC: total cholesterol; TG: triglycerides; WHR: waist-to-hip ratio.

## Competing interests

The authors declare that they have no competing interests.

## Authors' contributions

The authors' responsibilities were as follows--CRD conducted the statistical analysis, interpretation of results, and drafting of the manuscript; DP, BIG, LMF, and RS contributed to the interpretation of results and drafting of the manuscript; KK served as the study nutritionist and with RS developed the diet assessment; BIG provided statistical and methodological support; ND and PSG recruited study participants and acquired data; LR conducted laboratory analysis; HS and AM provided site-specific dietary assessment support; YS provided data management and study coordination; RS, DP, AM, PCG, NC, TR, KR conceived of the study, and participated in its design and coordination. All authors read and approved the final manuscript.

## Supplementary Material

Additional file 1 **Appendix**. Distribution of participant characteristics across extreme tertiles of regional dietary patterns, India Health Study Click here for file
