# Supplementary material for: A cross-sectional investigation of regional patterns of diet and cardio-metabolic risk in India
Source: Nutr J. 2011 Jan 28;10:12. doi: 10.1186/1475-2891-10-12 (PMC3042918; doi:10.1186/1475-2891-10-12)
Supplement: Additional file 1 — Appendix. Distribution of participant characteristics across extreme tertiles of regional dietary patterns, India Health Study [file 1475-2891-10-12-S1.DOC]

| Appendix. Distribution of participant characteristics across extreme tertiles of regional dietary patterns, India Health Study | | | | | | | | | | | | | | | | | | | | | | | | | | | | | | | | | | | | | | | | | | | | | | | | | | | | | | | | | | | | | | | | | | | | |  | | | | | |
| --- | --- | --- | --- | --- | --- | --- | --- | --- | --- | --- | --- | --- | --- | --- | --- | --- | --- | --- | --- | --- | --- | --- | --- | --- | --- | --- | --- | --- | --- | --- | --- | --- | --- | --- | --- | --- | --- | --- | --- | --- | --- | --- | --- | --- | --- | --- | --- | --- | --- | --- | --- | --- | --- | --- | --- | --- | --- | --- | --- | --- | --- | --- | --- | --- | --- | --- | --- | --- | --- | --- | --- | --- | --- | --- |
|  | | Delhi *(n=824)* | | | | | | | | | | | | | | | | | | | Trivandrum *(n=2,247)* | | | | | | | | | | | | | | | | | | | | | | | | | | | | | | | Mumbai *(n= 743)* | | | | | | | | | | | | | | | | | |  | | | | |
|  | | Factor 1 | | | | | | | Factor 2 | | | | | | | | | | | | | Factor 1 | | | | | | | | | | | | Factor 2 | | | | | | | | | | | | | | | | Factor 1 Factor 2 | | | | | | | | | | | | | | | | | | | | | | | | |
|  | | *fruit - dairy* | | | | | | | *veg - pulses* | | | | | | | | | | | | | *pulses - rice* | | | | | | | | | | | | *sweets - snacks* | | | | | | | | | | | | | | | | *fruit – veg snacks - meat* | | | | | | | | | | | | | | | | | | | | | | | | |
| Characteristic | | T1 | | | T3 | | | | T1 | | | | | T3 | | | | | | | T1 | | | | | T3 | | | | | | | T1 | | | | | | | | | T3 | | | | | | | | T1 | | | | | | | | | T3 | | | T1 | | | | T3 | | | | | |  | | |
| Dietary pattern score^*^, f / m^**^ | | -0.7/-0.7 | | | 0.7/0.6 | | | | -0.9/-0.9 | | | | | 1.1/1.1 | | | | | | | -0.9/-0.9 | | | | | 0.9/0.9 | | | | | | | -1.0/-0.9 | | | | | | | | | 0.9/0.9 | | | | | | | | -0.6/-1.3 | | | | | | | | | 0.6/1.2 | | | -1.1/-0.7 | | | | 1.1/0.7 | | | | | |  | | |
| Age, years^*^ | | 44 | | | 46 | | | | 45 | | | | | 48 | | | | | | | 47 | | | | | 51 | | | | | | | 50 | | | | | | | | | 47 | | | | | | | | 51 | | | | | | | | | 50 | | | 50 | | | | 49 | | | | | |  | | |
| Female, % | | 54 | | | 54 | | | | 54 | | | | | 54 | | | | | | | 50 | | | | | 50 | | | | | | | 50 | | | | | | | | | 50 | | | | | | | | 53 | | | | | | | | | 53 | | | 53 | | | | 53 | | | | | |  | | |
| Middle school graduate, % | | 47 | | | 27 | | | | 28 | | | | | 38 | | | | | | | 42 | | | | | 51 | | | | | | | 54 | | | | | | | | | 37 | | | | | | | | 50 | | | | | | | | | 29 | | | 40 | | | | 35 | | | | | |  | | |
| 2⁰ school graduate, % | | 26 | | | 28 | | | | 29 | | | | | 31 | | | | | | | 51 | | | | | 36 | | | | | | | 40 | | | | | | | | | 48 | | | | | | | | 48 | | | | | | | | | 64 | | | 57 | | | | 58 | | | | | |  | | |
| University graduate, % | | 27 | | | 45 | | | | 44 | | | | | 31 | | | | | | | 7 | | | | | 13 | | | | | | | 6 | | | | | | | | | 14 | | | | | | | | 2 | | | | | | | | | 7 | | | 3 | | | | 7 | | | | | |  | | |
| Married, % | | 93 | | | 93 | | | | 96 | | | | | 92 | | | | | | | 93 | | | | | 92 | | | | | | | 94 | | | | | | | | | 93 | | | | | | | | 77 | | | | | | | | | 86 | | | 81 | | | | 82 | | | | | |  | | |
| Low income, % | | 10 | | | 3 | | | | 5 | | | | | 5 | | | | | | | 72 | | | | | 68 | | | | | | | 76 | | | | | | | | | 64 | | | | | | | | 47 | | | | | | | | | 22 | | | 38 | | | | 28 | | | | | |  | | |
| Middle income, % | | 23 | | | 16 | | | | 8 | | | | | 23 | | | | | | | 25 | | | | | 27 | | | | | | | 21 | | | | | | | | | 32 | | | | | | | | 41 | | | | | | | | | 51 | | | 48 | | | | 48 | | | | | |  | | |
| High income, % | | 68 | | | 80 | | | | 87 | | | | | 71 | | | | | | | 3 | | | | | 5 | | | | | | | 3 | | | | | | | | | 4 | | | | | | | | 12 | | | | | | | | | 27 | | | 14 | | | | 24 | | | | | |  | | |
| Hindu, % | | 84 | | | 72 | | | | 72 | | | | | 79 | | | | | | | 18 | | | | | 53 | | | | | | | 27 | | | | | | | | | 44 | | | | | | | | 57 | | | | | | | | | 62 | | | 59 | | | | 60 | | | | | |  | | |
| Muslim, % | | 4 | | | 3 | | | | 5 | | | | | 1 | | | | | | | 42 | | | | | 24 | | | | | | | 35 | | | | | | | | | 33 | | | | | | | | 0 | | | | | | | | | 0 | | | 0 | | | | 0 | | | | | |  | | |
| Christian, % | | 0 | | | 1 | | | | 0 | | | | | 1 | | | | | | | 40 | | | | | 23 | | | | | | | 39 | | | | | | | | | 24 | | | | | | | | 2 | | | | | | | | | 8 | | | 3 | | | | 5 | | | | | |  | | |
| Other primary religion, % | | 12 | | | 25 | | | | 23 | | | | | 19 | | | | | | | 0 | | | | | 0 | | | | | | | 0 | | | | | | | | | 0 | | | | | | | | 41 | | | | | | | | | 31 | | | 38 | | | | 35 | | | | | |  | | |
| Never use tobacco, % | | 77 | | | 84 | | | | 83 | | | | | 82 | | | | | | | 72 | | | | | 74 | | | | | | | 72 | | | | | | | | | 72 | | | | | | | | 41 | | | | | | | | | 54 | | | 45 | | | | 50 | | | | | |  | | |
| Never consume alcohol, % | | 78 | | | 79 | | | | 82 | | | | | 80 | | | | | | | 82 | | | | | 87 | | | | | | | 86 | | | | | | | | | 85 | | | | | | | | 76 | | | | | | | | | 80 | | | 85 | | | | 70 | | | | | |  | | |
| Physical activity, %^**^ |  | | | | | | |  | | | | |  | | | | |  | | | |  | | |  | | | | | |  | | | | | | | | |  | | | | | | | |  | | | | | |  | | | | | |  | | | |  | | |  | | | | | | | |
| T1 (low) | | 22 | | | 50 | | | | 42 | | | | | | | | 26 | | | | 25 | | | | | | | | 45 | | | | | | | | 43 | | | | | | | | 27 | | | | | 47 | | | | | | | 24 | | | | | 27 | | | | | 42 | | | | | |  | |
| T3 (high) | | 46 | | | 16 | | | | 25 | | | | | | | | 34 | | | | 40 | | | | | | | | 25 | | | | | | | | 26 | | | | | | | | 38 | | | | | 17 | | | | | | | 46 | | | | | 39 | | | | | 26 | | | | | |  | |
| Total energy intake, kcal/day^*^ | | 2,579 | | | 2,226 | | | | 2,213 | | | | | | | | | 2,586 | | | 3,036 | | | | | | | 1,937 | | | | | | | | | | 2,138 | | | | | | | | 2,723 | | | | 2,780 | | | | | | | 4,034 | | | | | 2,854 | | | | | 3,618 | | | | | |  | |
| Abdominal adiposity, % | | 86 | | | 96 | | | | 92 | | | | | 87 | | | | | | | 91 | | | | | | 88 | | | | | | | | 85 | | | | | | | | | 90 | | | | | | 79 | | | | | | | 87 | | | | | 80 | | | | | 89 | | | | | |  | |
| Hypertension, % | | 38 | | | 62 | | | | 53 | | | | | 49 | | | | | | | 51 | | | | | | 54 | | | | | | | | 53 | | | | | | | | | 49 | | | | | | 52 | | | | | | | 43 | | | | | 43 | | | | | 51 | | | | | |  | |
| IFG > 100, % | | 53 | | | 56 | | | | 56 | | | | | 55 | | | | | | | 53 | | | | | | 54 | | | | | | | | 51 | | | | | | | | | 56 | | | | | | − | | | | | | − | | | | | | − | | | | | − | | | | | |  | |
| Diabetes, % | | 17 | | | | 15 | | | | 18 | | | | 18 | | | | | | | 28 | | | | | | | | 31 | | | | | | 28 | | | | | | | 29 | | | | | | | | 11 | | | | | | | | 10 | | | | 13 | | | | 6 | | | | | | |  | |
| Dyslipidemia, % | | 57 | | | | 56 | | | | 55 | | | | 53 | | | | | | | − | | | | | | | | − | | | | | | − | | | | | | | − | | | | | | | | − | | | | | | | | − | | | | − | | | | − | | | | | | |  | |
| Dietary intake, % of TEI^*^ | |  | | | |  | | |  | | | | |  | | | | | |  | | |  | | | | | | |  | | | | | | | | |  | | | | | | | |  | | | | | |  | | | | | | | | |  | | |  | | | | | | | |  | |
| Fat | | | 42 | | | | 40 | | | | 39 | | | | 42 | | | | | | 21 | | | | | | | 25 | | | | | | | | 20 | | | | | | | 26 | | | | | | | | 39 | | | | | | | 39 | | | | | 40 | | | | 39 | | | | | |  | |
| Carbohydrate | | | 48 | | | | 50 | | | | 51 | | | | 48 | | | | | | 63 | | | | | | | 59 | | | | | | | | 63 | | | | | | | 58 | | | | | | | | 47 | | | | | | | 48 | | | | | 48 | | | | 47 | | | | | |  | |
| Protein | | | 12 | | | | 12 | | | | 12 | | | | 12 | | | | | | 15 | | | | | | | 15 | | | | | | | | 15 | | | | | | | 15 | | | | | | | | 14 | | | | | | | 13 | | | | | 13 | | | | 14 | | | | | |  | |
| ^*^Presented as median value | | | |  | | | | | | | |  | | | |  | | |  | | | | |  | | | | | | | |  | | | | | | | | |  | | | | | | | |  | | | | | |  | | | | | |  | | | | | | |  | | |  | | |  |
| ^**^ Region and sex-specific cutpoints | | | | | | | | | | | |  | | | |  | | |  | | | | |  | | | | | | | |  | | | | | | | | |  | | | | | | | |  | | | | | |  | | | | | |  | | | | | | |  | | |  | | | |
